# Multi-Therapeutic Potential of Naringenin (4′,5,7-Trihydroxyflavonone): Experimental Evidence and Mechanisms

**DOI:** 10.3390/plants9121784

**Published:** 2020-12-16

**Authors:** Azher Arafah, Muneeb U. Rehman, Tahir Maqbool Mir, Adil Farooq Wali, Rayeesa Ali, Wajhul Qamar, Rehan Khan, Ajaz Ahmad, Syed Sameer Aga, Saeed Alqahtani, Nada M. Almatroudi

**Affiliations:** 1 Department of Clinical Pharmacy, College of Pharmacy, King Saud University, P.O. Box 2457, Riyadh 11451, Saudi Arabia; aazher@ksu.edu.sa (A.A.); aajaz@ksu.edu.sa (A.A.); saeed@ksu.edu.sa (S.A.); 2 Division of Veterinary Biochemistry, Faculty of Veterinary Science and Animal Husbandry, SKUAST-Kashmir, Alustang, Shuhama 190006, India; alirayeesa@gmail.com; 3 National Center for Natural Products Research, Research Institute of Pharmaceutical Sciences, School of Pharmacy, University of Mississippi, University, MS 38677, USA; tmmir@olemiss.edu; 4 Department of Pharmaceutical Chemistry, RAK College of Pharmaceutical Sciences, RAK Medical and Health Science University, Ras Al Khaimah 11171, UAE; 5 Department of Pharmacology & Toxicology, College of Pharmacy, King Saud University, P.O. Box 2457, Riyadh 11451, Saudi Arabia; wqidris@ksu.edu.sa; 6 Department of Nano-Therapeutics, Institute of Nano-Science and Technology (DST-INST), Mohali 160047, India; rehankhan@inst.ac.in; 7 Department of Basic Medical Sciences, College of Medicine, King Saud bin Abdulaziz University (KSAU-HS), King Abdullah International Medical Research Center (KAIMRC), Ministry of National Guard Health Affairs (NGHA), Jeddah 21423, Saudi Arabia; agas@ksau-hs.edu.sa; 8 Department of Clinical Pharmacy, College of Pharmacy Girls Campus, King Saud University, P.O. Box 2457, Riyadh 11451, Saudi Arabia; 439204163@student.ksu.edu.sa

**Keywords:** naringenin, pharmacology, natural medicine, phytochemistry, polyphenols

## Abstract

Extensive research has been carried out during the last few decades, providing a detailed account of thousands of discovered phytochemicals and their biological activities that have the potential to be exploited for a wide variety of medicinal purposes. These phytochemicals, which are pharmacologically important for clinical use, primarily consist of polyphenols, followed by terpenoids and alkaloids. There are numerous published reports indicating the primary role of phytochemicals proven to possess therapeutic potential against several diseases. However, not all phytochemicals possess significant medicinal properties, and only some of them exhibit viable biological effects. Naringenin, a flavanone found in citrus fruits, is known to improve immunity, repair DNA damage, and scavenge free radicals. Despite the very low bioavailability of naringenin, it is known to exhibit various promising biological properties of medicinal importance, including anti-inflammatory and antioxidant activities. This review focuses on the various aspects related to naringenin, particularly its physicochemical, pharmacokinetic, and pharmacodynamic properties. Furthermore, various pharmacological activities of naringenin, such as anticancer, antidiabetic, hepatoprotective, neuroprotective, cardioprotective, nephroprotective, and gastroprotective effects, have been discussed along with their mechanisms of action.

## 1. Introduction

Natural products are a rich source of phytochemicals, including polyphenols, which provide a pool of antioxidants for the maintenance of steady health. Polyphenols are categorized based on the structure of phenol rings and functional moieties that interconnect these phenolic rings and hence are divided into flavonoids, lignans, stilbenes, and phenolic acids. Flavonoids, which are mainly natural pigments, are primarily found in plants. They are comprised of a basic phenolic structure [1,2] and have secured a place in folk medicine globally due to their beneficial biological effects [3]. Among over 4000 flavonoids that have been discovered to date, several have been found to exhibit a wide variety of beneficial biological activities. Among them, naringenin has gained much attention lately, as many studies have suggested that several of its biological properties are of medicinal importance [4,5].

**Figure 1:**
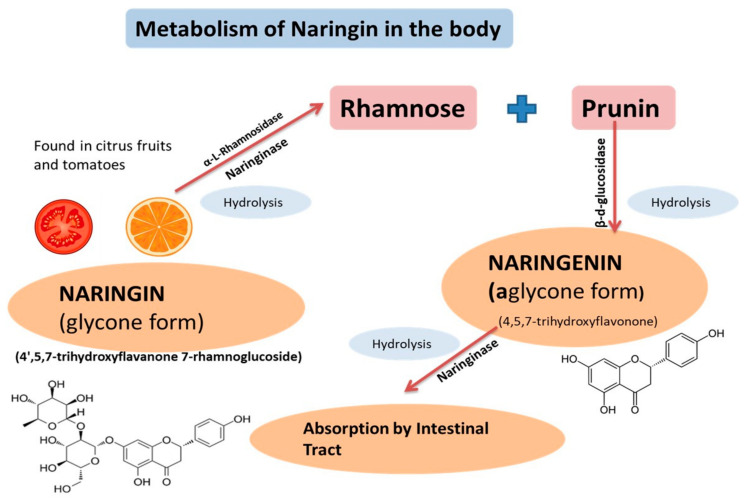
Naringin is hydrolyzed by naringinase and α-L-rhamnosidase into prunin and rhamnose. With the help of β-d-glucosidase, prunin then is hydrolyzed into “Naringenin,” which is absorbed into the intestinal tract after being hydrolyzed by naringinase.

Naringenin (4′,5,7-trihydroxyflavonone) is mainly found in citrus fruits (grapefruit and oranges) and tomatoes. It is synthesized from an aromatic amino acid, phenylalanine, and has been found in different conjugated forms, primarily as aglycone, neohesperidoside, and glycosylated forms. Each of these forms differs in its pharmacokinetic (absorption, distribution, metabolism, and elimination) properties. The richest source of naringenin is grapefruits, where it is present as an inactive glycone form called “naringin.” Chemically, naringin is a 4′,5,7-trihydroxyflavonone 7-rhamnoglucoside that is hydrolyzed by the intestinal bacterial naringinase enzyme soon after its ingestion, resulting in the production of two intermediates, rhamnose and naringenin (4′,5,7-trihydroxyflavonone—the most active aglycone form) [6] (Figure 1). Naringenin has been reported to be easily absorbed by the intestinal tract; thus, it becomes rapidly bioavailable in circulation. Because of the rapid absorption, it is the most pharmacologically effective form of naringin [7].

Naringenin is known to have poor water solubility, which affects its overall bioavailability [8]. The pharmacological activities associated with naringenin have been attributed to its ability to suppress oxidative stress by scavenging free radicals generated during various basal metabolic conditions [9]. Presently, attempts are made for the de novo synthesis of pharmacologically numerous active flavonoids, including naringenin from *Escherichia coli*, in order to ensure their cost-effective high production [10]. In this article, we attempt to review the available scientific literature on various biological and pharmacological properties of naringenin. We exploited two largely available scientific databases, PubMed and Google Scholar, for literature search. The literature was searched using keywords, such as “polyphenols”, “flavonoids”, “aglycone”, “naringin”, “naringenin”, “sources of naringenin”, “forms of naringenin”, “structure activity relationship for naringenin”, “bioavailability of naringenin”, “pharmacokinetics of naringenin”, “pharmacodynamics of naringenin”, “in vitro and in vivo pharmacological properties of naringenin such as anti-cancer, anti-diabetic”, “hepatoprotective”, “neuro-protective”, “cardio-protective”, “nephron-protective”, and “gastro-protective and immune-modulatory effects”. Only a few clinical trials on naringin or naringenin were registered at clinicaltrials.gov [11], indicating that tremendous efforts are required for the transition of this natural product from preclinical to clinical use. The information from different research reports was collected and presented in different relevant sections and subsections of this review.

## 2. Physicochemical Characteristics, Pharmacokinetics, and Pharmacodynamics of Naringenin

Naringenin has a simple 15-carbon-atom flavonoid skeleton, which comprises three rings. Among them, two benzene rings are linked to a three-carbon chain [12]. The chemical nomenclature of naringenin is 2,3-dihydro-5,7-dihydroxy-2-(4-hydroxyphenyl)-4H-1-benzopyran-4-one or 4′,5,7-trihydroxyflavanone, with a molar mass of 272.3 [13]. The major mechanism by which naringenin exhibits its diverse bioactivities is through the suppression of oxidative stress, which is caused by various free radical species produced in the organisms as byproducts of aerobic metabolism. The existence of the 2, 3-double bond linked to the 4-oxo group of naringenin has been demonstrated to be a critical functional active group responsible for its antioxidant potential [12,14].

Naringenin has a very low affinity for water with a water solubility near 46 ± 6 µg/mL, which limits its oral bioavailability to approximately ~5.81%. It undergoes rapid hepatic first-pass metabolism and is transformed into glucuronide intermediary products, resulting in its limited bioavailability in plasma. Sun et al. reported that the t_1/2_ of naringenin is about 4.69 h, along with C_max_ = 2910.6 ng/mL and area under curve (AUC)  =  40,607.9 ng/mL after oral ingestion (Zhi Zhu Wan, a traditional Chinese medicine) [15]. Kanaze et al. reported that the absorption of naringenin reached a peak after 3.5 h of oral consumption in human subjects (135 mg), with Cmax = 2009.51 +/− 770.82 ng/mL and AUC (0-infinity) = 9424.52 +/− 2960.52 ng h/mL [16]. The authors suggested that, despite its fast absorption, the bioavailability of naringenin remained low due to extensive first-pass metabolism in the intestine. After oral intake of naringin or grapefruit juice, significant concentrations of naringenin have been found in peripheral blood and urine [8]. Recently, researchers have utilized different techniques and complex inclusion methods to develop numerous viable naringenin formulations. These techniques include liposomes, nanoparticles, self-nano-emulsifying drug delivery systems (SNEDDS), and nanosuspensions to improve its bioavailability, which would enhance its clinical applications [17,18].

**Figure 2:**
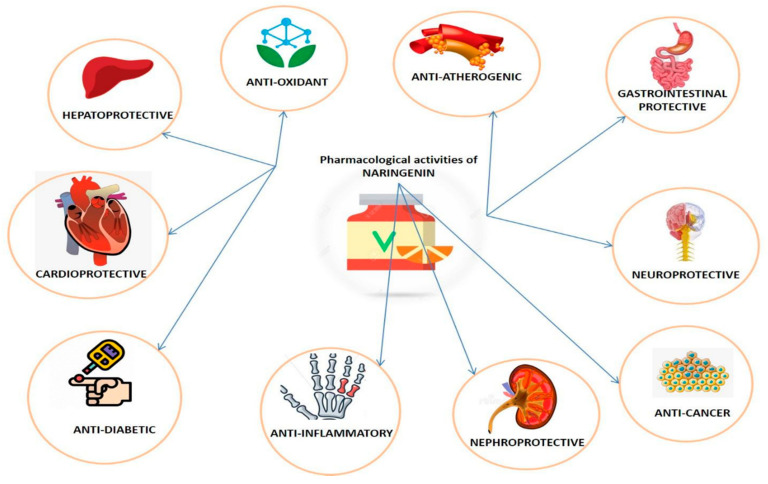
Different pharmacological activities of Naringenin.

Naringenin is relatively safe with LD50 of 5000 mg/kg [19]. The chief mechanism of action of naringenin is the inhibition of specific cytochrome P450 isoforms—CYP1A2 [20] and CYP3A4 in humans [21]. Naringenin has successfully demonstrated antagonistic activities on all types of opioid receptors [22]. It has positive health effects in humans; concurred by numerous research reports indicating that it has hepatoprotective [23], anti-mutagenic [24], anti-carcinogenic [25], antioxidant [26], anti-diabetic [27], and antiatherogenic [28] activities (Figure 2). The daily dietary requirement of flavonoids has been considered to be a few hundred milligrams [29]. Its role against inflammation and oxidative injuries makes it a potential candidate for the treatment of various oxidative-stress-related disorders [22,23,24,25,26,27,28,29].

## 3. Pharmacological Properties of Naringenin

### 3.1. Anticancer Properties

**Figure 3:**
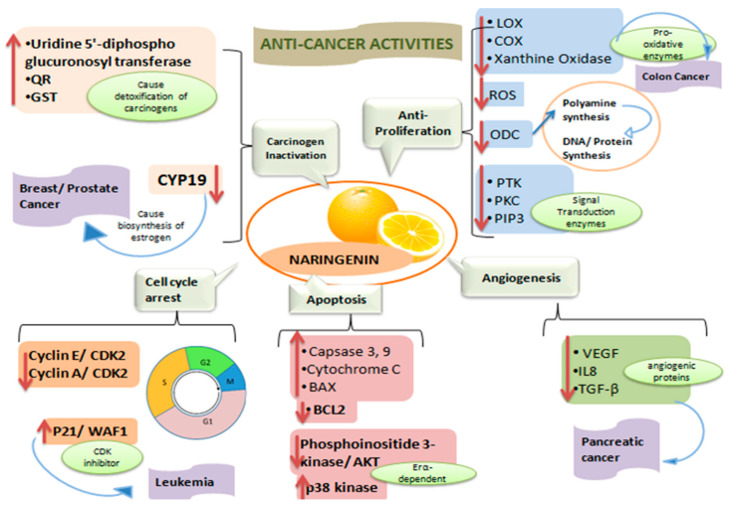
Naringenin found in citrus fruits is a flavonoid that has anti-cancer properties.

Cancer is one of the leading causes of death worldwide [30]. Mutations of DNA, caused by any exogenous stimulus or an endogenous anomaly, in normal cells can result in the uncontrolled division of cells, leading to tumor development and progression. Chemotherapeutic agents appear to exert anti-cancerous activity by assisting in cell apoptosis [31]. However, the devastating effects of the chemotherapy on the living system are colossal; i.e., every healthy cell and primarily rapidly dividing cells, including gastric mucosa, hair follicle, and bone marrow cells, are deleteriously affected in this process, which restricts their normal functioning [32]. Therefore, toxicity appears to be the most limiting feature associated with the chemotherapeutic agents of synthetic origin. On the other hand, therapeutically active plant-derived products/components are considered minimally toxic while having significant pharmacological attributes [33,34]. Globally, the anti-cancer potential of flavonoid “naringenin” has been widely debated [25]. The case-controlled study conducted by Stefani et al. in Uruguay has reported an approximately 70% decrease in cancer risk associated with the esophagus, larynx, oral cavity, and pharynx after naringenin administration [35] (Figure 3).

The appraisal discussed below of naringenin can help in better understanding the molecular mechanisms behind its anti-cancer activities.

Naringenin causes carcinogen inactivation by upregulation of the Uridine 5’-diphospho glucuronosyltransferase, QR, and GST that help in the removal of carcinogens in the body. By inhibition of CYP19, it helps to control breast and prostate cancer. It has anti-proliferative properties exhibited by downregulation of ROS and ODC, and signal transduction enzymes like PTK, PKC, and PIP3 decrease the unwanted proliferation of cells. Naringenin causes cell cycle arrest in G and S phases in cancer cells by inhibiting cyclin, and CDK helps to control leukemia. The increase in caspase, cytochrome, and BAX and the downregulation of BCL2 in mitochondria are caused by naringenin, and the inhibition of ERα-dependent mitogenic signaling cascades activation (e.g., phosphoinositide 3-kinase/AKT) or by induction of ERα-dependent p38 kinase activation causes pro-apoptotic activities in cancer cells. Naringenin exhibits its angio-inhibitory effect by decreasing vascular endothelial growth factor (VGFG) and downregulating the TGF-β pathway, thereby decreasing metastasis and invasion in pancreatic cells.

#### 3.1.1. Carcinogen Inactivation

Human enzymes make a significant contribution to the metabolic conversion of pro-carcinogens into active carcinogens. This transformation is largely executed via cytochrome P450. Naringenin antagonizes this conversion and therefore prevents carcinogenesis and damage to the cells [36]. Naringenin is also known to inhibit the activity of enzyme aromatase (CYP19), which leads to a decrease in the biosynthesis of estrogen. The anti-estrogenic effects so produced may prove to be of significant anti-prostate- and anti-breast-cancer potential [37]. In estrogen-dependent breast cancer, naringenin has exhibited inhibitory effects on estrogen synthetase enzyme (aromatase), which is involved in the biosynthesis of estrogen, thereby reducing cancer-promoting stimulatory effects of estrogens [38,39].

#### 3.1.2. Anti-Proliferative Action

The mechanism of carcinogenesis takes place in two distinct stages: initiation and progression. The progression stage of carcinogenesis involves products of several genes (especially tumor suppressors) [40], which can serve as specific targets for naringenin to prevent or stop cancer development. Investigations have revealed that the generation of ROS (reactive oxygen species) is one of the key mechanisms responsible for promoting different stages of cancer [41]. Naringenin, as an antioxidant, does efficiently counter such effects. Additionally, restriction to the formation of precancerous lesions via regulation of hyperproliferation has also been reported as one of the bio-effects of naringenin [42].

Anti-proliferative action of flavonoids has been demonstrated in numerous in vitro studies using various kinds of cancer cells [43,44]. Naringenin inhibits xanthine oxidase [45] cyclooxygenase, lipoxygenase [46] and hampers the proliferation of tumor cells. In experimentally induced colon cancer, naringenin has been reported to inhibit hyperproliferation. Inhibition of ornithine decarboxylase (ODC) activity, in several tissues, by naringenin has also been reported. ODC is involved in the biosynthesis of polyamine, and thus inhibited ODC activity results in the consequent decrease in polyamine and reduced DNA/protein synthesis [47]. Furthermore, naringenin has shown inhibitory effects on signal transduction enzymes, playing an important role in controlling cell growth by regulating proteins like protein tyrosine kinase (PTK) [48,49] protein kinase C (PKC) [50], and phosphoinositide 3-kinases (PIP3) [51]. N-nitrosodiethanolamine (NDEA)-induced hepatocarcinogenic rats after pre- and post-treatment with naringenin have revealed a decline in proliferating cell nuclear antigen (PCNA), which is one of the important markers of hyperproliferation [52,53,54]. Naringenin has been found to suppress the rate of PCNA expression in leukemia cell line HL-60, thereby indicating its anti-proliferative activity [55].

#### 3.1.3. Cell Cycle Arrest

In response to growth-stimulating signals, cells progress through the various distinct stages of the cell cycle like G1, S, G2, and M, each of which is regulated by activated cyclin-dependent kinases (CDKs). CDKs are activated after adhering to a family of proteins called cyclins. For each phase of the cell cycle, there are diverse stage-specific groups of cyclins and CDKs. There are two families of CDK inhibitors, including p21, p27, and p57, which regulate the cell cycle. Mutation in any of the cell-cycle-regulating genes results in either overexpression or dysregulation of cyclins/CDKs, thus assisting the progression of cancerous cells into the uncontrolled mass of dividing cells. In vitro studies have revealed that naringenin exhibited anti-cancer activity by arresting the cell cycle at the G0/G1 phase along with the induction of apoptosis [56]. It has been also shown to arrest the growth in K562 human leukemia cell line, particularly in the G0/G1 phase of the cell cycle. The inhibition may be achieved via upregulation of p53-independent p21/WAF1. Suppression of cyclin E/CDK2 and Cyclin A/ CDK2 has also been reported [52].

#### 3.1.4. Induction of Apoptosis

Cancers arise due to dysregulated apoptosis in addition to the change of other pathways like upregulation of growth-promoting oncogenes and downregulation of tumor suppressor genes. Similarly, cell apoptosis, like cell growth, is also tightly regulated by certain promoter and inhibitor genes. Apoptosis does involve several genes for two specific pathways, including intrinsic (*BAX, BAK, APAF-1,* and caspase family) and extrinsic (*TRAIL, TRADD, FADD, FAS,* etc.) pathways. Activation of caspases such as 3 and 9, following release of cytochrome-c, happens to be the pathway that flavonoids trace for inducing apoptosis [57]. Naringenin is believed to inhibit unregulated growth in different cancer cell types executing apoptosis, such as in K562 cell line containing ERα or ERβ receptors [58,59]. The pro-apoptotic action on cell lines expressing ERα is either by the inhibition of ERα-dependent mitogenic signaling cascade activation (e.g., phosphoinositide 3-kinase/AKT) or by induction of ERα-dependent p38 kinase activation (a member of the mitogen activating protein kinase family) [58,60]. The possible mechanisms behind the programmed cell death may be the downregulation of Bcl-2 and the upregulation of Bax and Caspase activity [52]. Western blot analysis of the naringenin-treated cells has shown an enhanced concentration of pro-apoptotic protein Bad and a diminished concentration of anti-apoptosis proteins like Bcl-2 [61]. It has been also found that naringenin modulates cellular membrane biophysical parameters such as membrane fluidity, membrane protein and bilayer structures, lipid molecule packaging, and hydration and thereby induces mitochondrial potential and promotes apoptotic activity [62].

#### 3.1.5. Inhibition of Sustained Angiogenesis

For tumor progression, angiogenesis or neovascularization is a crucial step as it provides essential nutrient supply to the transformed cells. Equilibrium between angiogenic and anti-angiogenic proteins act as a barrier for tumor growth and progression. Angiogenic proteins include VEGF, basic fibroblast growth factor, IL8, and TGF-β [63,64], whereas anti-angiogenic factors include thrombospondin-1, angiostatin, and endostatin. Flavonoids such as naringenin act as effective inhibitors of angiogenesis and hence are regarded as a good treatment for the control of malignancies [65]. Naringenin exhibits its angio-inhibitory effect by decreasing vascular endothelial growth factor (VEFG) and other related factors [66]. It has also been reported to cause downregulation of TGF-β pathway, thereby decreasing metastasis and invasion in pancreatic cells [67].

### 3.2. Anti-Diabetic Activity

Diabetes is a metabolic disorder of endocrine origin identified by elevated blood glucose levels. It has been estimated to be the major cause of global morbidities and mortalities linked to diabetes-related afflictions [68]. The disease has two major types, i.e., type 1 and type 2. The former is caused by inadequate or no insulin production, and the latter is caused by insulin deficiency and/or relative insufficiency because of β cell abnormality and compromised response to insulin [68].

Hyperglycemia, or high blood glucose levels in the body, is the primary indication of diabetes. Glucose and maltose are the catabolic byproducts of starch [69], which are in turn catalyzed by α-glucosidase and α-amylase. It has been reported that inhibition of the activity of these key enzymes can result in the decreased levels of blood glucose. Utilization of these enzyme targets can serve as potential remedies for improving diabetes-related disorders. Naringenin has been found to possess effective anti-diabetic potential, which is attributed to its ability to inhibit α-glucosidase and α- amylase [70]. Scientific evidence reports that naringenin administration results in the decline of blood glucose levels in streptozotocin-induced diabetic rats [12,71], develops insulin sensitivity in insulin-resistant rats that were given fructose [72], and diminishes resistance to insulin in mice consuming high-fat diets that lacked LDL receptors [73]. The mechanism of action of naringenin in type 2 diabetics is comparable to that of conventional antidiabetic metformin [74].

The adipose tissues of diabetic and obese people have exhibited a rich expression of numerous TLR family members [75]. Activation of TLR2 and TLR4 receptors by free fatty acids (FFA) ends up as insulin resistance [76,77]. Naringenin inhibits TLR2 expression in adipocytes through activation of peroxisome proliferator-activated receptor-gamma (PPARγ) [78]. It also downregulates TNF-α-induced TLR2 expression by decreasing NF-κB and c-Jun NH2-terminal kinase (JNK) pathways in differentiated adipocytes. Further pathology behind the advancement of insulin resistance and diabetes mellitus is the interaction of adipocytes and macrophages in the adipose tissue, which results in the inflammation of adipose tissue. Naringenin counteracts this inflammation by suppression of monocyte chemoattractant protein-1 (MCP-1) and migration of macrophages into adipose tissue [79]. Of all biomolecules, lipids have an inevitable role in the pathogenesis of diabetes mellitus [80]. A common metabolic anomaly related to diabetes mellitus is dyslipidemia. Research findings have reported various abnormalities in lipid metabolism attended by the risk of cardiovascular arteriosclerosis in DM patients [81]. Naringenin has been reported to have insulin-like effects, according to in vitro studies, which increase the expression of LDL receptor via PI3K-mediated upregulation of SREBP-1 in the cytosol and nucleus of human HepG2 and rat McA-RH7777 hepatoma cells. Naringenin treatment escalates the PI3K activity and reduces insulin receptor substrate-2 (IRS-2) levels. The plausible mechanism for naringenin’s insulin-like effects is probably the increased activity of PI3K [82].

**Figure 4:**
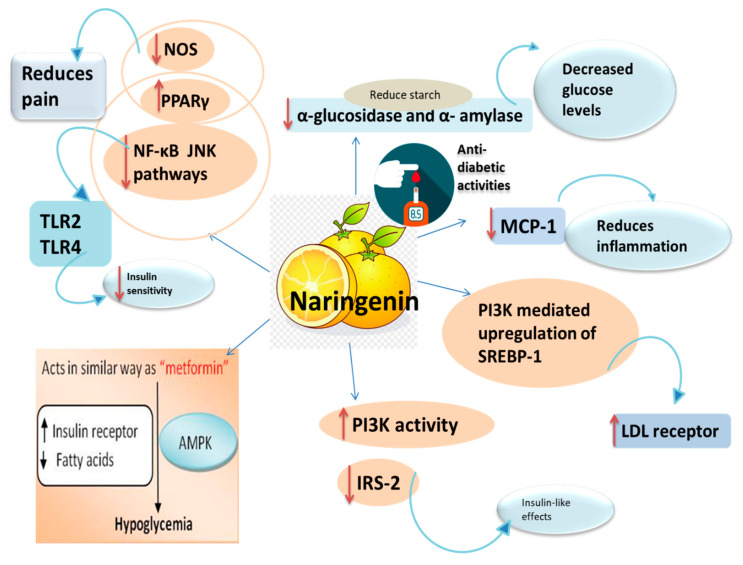
Different mechanisms involved in metabolic disorders such as diabetes and the role of naringenin in the regulation of several pathways involved in metabolic syndrome.

Diabetic neuropathic pain is one of the chief complications associated with diabetes mellitus [83]. Afflicted individuals often have complaints of “shock-like ambiances” with amplified sensations to external stimuli. This can have harmful and disabling effects [84,85]. Naringenin-treated Wistar rats have shown reduced pain and allodynic effects of diabetic neuropathy induced by streptozotocin [86]. Naringenin has also exhibited analgesic effects in an experimental model of neuropathic pain [87]. The possible mechanism behind the beneficial effects of naringenin may be the trigger of PPARγ [88]. Several studies have revealed that naringenin exhibits agonistic activity at the PPARγ receptor and therefore ameliorates the pain in different nerve injuries and neuropathies [76]. Moreover, naringenin has been reported to cause inhibition of nitric oxide (NO) pathway via downregulation of nitric oxide synthase (NOS), which is the main enzyme for NO synthesis [89] and a neurotransmitter for nociception [90]. The anti-diabetic mode of action of naringenin is diagrammatically represented in Figure 4.

### 3.3. Hepatoprotective Action

The liver is a primary organ in the metabolism and detoxification of drugs or chemical entities; therefore, it is highly prone to being negatively affected. The increased exposure to drugs, viruses, and toxins triggers severe liver damage and intensifies the probability of chronic liver disease [91,92]. Production of reactive oxygen species (ROS) may be the primary reason behind hepatotoxicity that can lead to various liver diseases. ROS induces peroxidation of cell membranes and prompts cell damage that culminates in leaching of enzymes like aspartate transaminase (AST) and alanine transaminase (ALT) [93]. Free radicals are also believed to be involved in the activation of caspase-3, -8, and -9, leading to the induction of apoptosis during hepatic impairment [94,95].

Various research groups have reported the hepatoprotective potential of naringenin [11,96,97,98,99,100,101], which is attributed to its potential in neutralizing the ROS via increased expression and activation of antioxidant enzymes [102,103] and the ability to amend gamma-glutamylcysteine synthetase (GGT) enzyme activity, which is involved in the GSH synthesis pathway [104,105,106]. Reportedly, pre-treatment with naringenin causes a decline in levels of AST and ALT, while antioxidant parameters, viz GPx, SOD, CAT, and GSH, have shown a significant increase [101,107]. The groups of rats intoxicated with CCl4 have shown repression of caspase-3 and -9 levels [108]. In a recently published article by Wu et al., the hepatoprotective action of naringenin has been attributed to its potential in inhibiting hepatic oxidative stress and inflammation [109].

Intake of drugs and production of metabolic toxins in the body can pave the way for the production and release of ROS [38], cytokines, and other inflammatory factors. This activates the Kupffer cells within the liver, thereby resulting in the destruction of hepatocytes [110,111,112]. Hepatic stellate cells (HSCs), which play a role in the formation of basement membranes by producing small amounts of extracellular matrix components like collagen type IV and laminin, when exposed to factors released from the stimulated Kupffer cells, lose their lipid level and then go through phenotypic alterations, proliferate, and produce excess amounts of extracellular matrix, thus resulting in liver fibrosis [8,113,114]. Naringenin has shown hepatoprotective and anti-fibrotic effects against dimethylnitrosamine (DMN)-mediated liver injury in rats, while oral consumption of naringenin has resulted in reduction of AST, ALT, and ALP and bilirubin levels. Furthermore, staining of liver tissue sections with Sirius red stain has revealed a reduction in collagen accumulation in the naringenin-treated rats. Naringenin has also been reported to reduce the activation of hepatic stellate cells [103]. The effect of naringenin on inflammatory changes in the liver triggered by high dietary cholesterol has been extensively studied and has been found to significantly decrease the release of pro-inflammatory mediators and downregulate the expression of TNF-α and MMP-2 and MMP-9, which contributes to reduced macrophage infiltration [115,116]. It also modulates the levels of necrotic inflammation by degrading extracellular matrix [117,118,119]. Additionally, naringenin has been shown to alleviate inflammation induced by hyperglycemia in streptozotocin-nicotinamide-induced diabetes mellitus in rats [117].

### 3.4. Neuroprotective Action

In comparison to other body tissues, nerve cells are extremely sensitive to oxidative insults for the following reasons. (1) Brain cells are enriched in metal ions and polyunsaturated fatty acids. With the progression of time and age, the metal ions are accumulated in the brain, thus facilitating the formation of oxidative species. On the other hand, polyunsaturated fatty acids are more liable to oxidation and therefore may be responsible for oxidative stress. (2) In comparison to other cells, neuronal cells highly rely on oxidative phosphorylation for their main source of energy. (3) There is a lack of defense mechanisms operating in brain because it contains very meager quantities of antioxidant enzymes. These factors provide distinct evidence that the oversensitivity of nerve cells towards the stress is initiated and instigated by ROS [118].

It is a well-established fact that oxidative stress is substantially enhanced during the aging process, which may play an important role in increasing the vulnerability of neuronal systems to neurodegenerative diseases such as Parkinson’s and Alzheimer’s [119]. For the past ten decades, a 6-OHDA induced model of substantia nigra damage has established its identity as a classical model for the study of Parkinsonism in lab animals [120]. It is known to produce changes similar to the pathological features of Parkinson’s disease (PD). The lesion induced by 6-OHDA in the nigrostriatal pathway leads to continuous depletion of dopaminergic neurons in the substantia nigra pars compacta (SNpc) thus increasing oxidative stress [121]. Naringenin is thought to have high permeability towards blood-brain barrier (BBB) as per various in vitro and in situ studies [122,123]. Pre-treatment of rats with naringenin has exhibited significant neuroprotection against 6-OHDA-induced toxicity [124]. The evidence in favor of neuroprotection is supported by the affinity of naringenin towards the striatum and cerebral cortex [121]. The rotenone-induced PD model has also reported promising results in the administration of naringenin, with significant restoration of motor skills, body weight, expression of parkin, DJ-1, tyrosine hydroxylase, and chromatin immunoprecipitation (ChIP) in the striatum and substantia nigra [125]. Among the disorders of CNS, Alzheimer’s disease holds the maximum potential of negatively affecting the capability of learning and memory. It is usually characterized by amplified institution of beta-amyloid (Aβ) plaques. Alterations induced due to age in monoaminergic neurotransmitters (serotonin, noradrenaline, and dopamine) with an increase in tryptophan hydroxylase and tyrosine hydroxylase activity have also been reported. The Alzheimer rat model replicated by Aβ-injection has revealed improvement in cognitive functions through alleviation of lipid peroxidation and apoptosis on treatment with naringenin [80]. Studies reveal that naringenin could prove helpful in reversing the cognitive loss associated with AD by upregulating insulin signaling in the rat brain [126,127].

Covalent combination of naringenin with lipoic acid results in the generation of a novel compound, namely “VANL-100”. The in vitro and in vivo models of ischemia/reperfusion have exhibited neuroprotection at very low concentrations of 2 × 10^−2^ µM (100-fold more potent). The reason could be the increased capability of nerve cells in the presence of “VANL-100” to hunt down free radicals [9]. Naringenin was found to be effective in the management of cerebral ischemia by reducing oxidative injuries and NF-κB-mediated inflammation [128]. In another study, it was reported that naringenin has protective effects in experimental ischemic stroke models with improvement in neurological insufficiency and brain swelling with a reduction in infarct size [129].

The increase in the concentration of free radicals in neuronal cells may inhibit Acetylcholinesterase (AChE) activity. Rats exposed to iron have exhibited diminution in AChE levels, whereas naringenin administration to iron-exposed rats revealed a significant restoration in AChE activity due to its effective antioxidant potential [130]. In addition, naringenin supplementation exhibits the restoration of expression of acetyl choline transferase, resulting in the amendment of learning and memory in the treated models [131]. Improvement in cholinergic and serotonergic transmission upon treatment with naringenin has also been reported [132]. Naringenin also has the potential to protect the integrity of cells by controlling the antioxidant defense systems—both enzymatic as well as non-enzymatic ones. Therefore, naringenin can have modulatory effects on neuronal cholinergic neurotransmission resting membrane potential, directly or indirectly [116].

The capability of naringenin to alter glucose homeostasis in the brain has been recently studied. This can pave the way for a better understanding of insulin-dependent and -independent mechanisms involved in experimental diabetes. Pre-treatment with naringenin in epileptic mice has revealed its anti-epileptic potential as it curbs different oxidative stress biomarkers with the improvement in behavioral parameters [4].

### 3.5. Cardioprotective Action

Free radicals and reactive oxygen species (ROSs) are byproducts of the basal aerobic metabolism of cells. However, excessive generation of ROSs does result in critical oxidative damage to the cellular components of the cells [133,134]. Numerous studies suggest that naringenin exhibits profound antioxidant effects against various oxidative insults, which has been reported to improve cardiac functionality even after injuries [135]. The preclinical and clinical evidence in favor of this has been supported by the findings of various research groups [92]. One such study conducted by a team of researchers from Cairo University, Egypt, reported that there was a restoration in various antioxidant enzymes including superoxide dismutase (SOD), glutathione-S-transferase (GST), and catalase (CAT) on pre-treatment with naringenin against doxorubicin-induced cardiotoxicity in male Swiss albino rats [136]. In this context, Han et al. (2008) later studied the pathway utilized by naringenin to protect cardiomyocytes from doxorubicin-induced cardiac toxicity [137] and found that the upregulation of several endogenous enzymes possessing antioxidant activities via extracellular regulated kinase 1/2 (ERK1/2) phosphorylation and Nrf2 activation and translocation into the nucleus likely had a key role in the protection of cardiomyocytes. The findings indicated that naringenin protects against cardiomyocyte apoptosis by expressing higher endogenous antioxidants enzyme (SOD, GST, and CAT) via phosphorylation of ERK1/2 and nuclear translocation of Nrf2 [137]. Subsequently, other researchers opened the horizons of its mechanism and noted that naringenin suppresses the generation of reactive species [45], increases the activities of antioxidant enzymes such as superoxide dismutase (SOD) and catalases, and reduces the phosphorylation of ERK1/2 and p38 MAPK. Moreover, it prevents the binding of angiotensin II to angiotensin binding receptors AT1 receptor and AT2 receptor, thereby suppressing the vascular smooth muscle cell proliferation [138].

Naringenin exhibited its cardioprotective effect by reducing myocardial infarct area, decreasing LDH in coronary effluent to increase regenerative capacity, and improving the retrieval of left ventricular function. Naringenin-treated myocardium has also exhibited an increase in SOD and a decrease in levels of MDA [139]. The useful outcomes of naringenin on the heart can be related to its potential to act on calcium-activated potassium channels. Naringenin restricts the build-up of calcium in the cardiac mitochondrial matrix. An amendment of functional parameters post-ischemia with a lowering of the lengthening interval of myocardial injury has also been observed. The inner mitochondrial membrane depolarization depends on the concentration of thallium, and the transmembrane drift of thallium (potassium-mimetic cation) has been reported in isolated rat cardiac mitochondria upon interaction with naringenin [140].

Pink grapefruit is a hybrid citrus fruit produced by cross-hybridization of sweet orange (*Clonorchis sinensis*) and pomelo (*Cucurbita maxima*). It is known to be rich in naringenin. It increases temporal cardiac repolarization dispersion and therefore prolongs repolarization. In patients with structural disorders of the myocardium, it may be of value because of its pro-arrhythmic actions [141]. In pressure over-loaded mice, treatment with naringenin improved left cardiac hypertrophy and interstitial fibrosis. Amendment of left ventricular functions has also been reported. The action mechanism is thought to be via inhibition of ERK, PI3K/Akt, and JNK signaling pathways [142]. Hydrogen peroxide (H_2_O_2_) is one of the key ROSs, produced endogenously in the respiratory cascade of mitochondria and as a byproduct of basal metabolism. There are several other organs, such as the endoplasmic reticulum and peroxisome, that are thought to be its primary site of production. Due to its potent oxidative property, H_2_O_2_ induces severe detrimental effects on cardiomyoblasts, which may be reversed by naringenin treatments. Synthesis of antioxidant enzymes (CAT, SOD, and nitric oxide) decreases stress-facilitated apoptosis and lipid peroxidation with subsequent increase GSH levels. Naringenin has also shown upregulation in the transcription of Akt and Nrf2 coupled with downregulation of Caspase-3 and NF-κB genes. It also prevents apoptosis in H9c2 cardiac myocytes through inactivation of caspase-3, -8, and -9 and decreases the expressions of apoptosis (mitochondrial pathway) regulating proteins of Bcl-2 such as Bax and Bak [143,144].

Furthermore, naringenin administration in HCD rats exhibits significant improvements in serum levels of nitric oxide, LDH, oxidative damage markers of lipids, and lipid profile [119]. Naringenin was found to reduce the chances of atherosclerosis by alleviating dyslipidemia and insulin resistance, thereby inhibiting the assembly of apolipoprotein B100. Studies have also shown that naringenin increases fatty acid oxidation and blocks cholesterol synthesis and esterification, which results in reduced hepatic triglycerides and decreases cholesteryl ester; thus, there is a limited secretion of VLDL [145].

### 3.6. Nephroprotective Action

As per a WHO report, about 35 million people are suffering from chronic kidney diseases (CKD) in developing and developed countries. Inflammatory cytokines and oxidative stress play a pivotal role in the progression of renal damage. The specific targets for renal injury are the mesangial cells, glomerular endothelial cells, podocytes, and tubular epithelial cells [146]. Other forms of kidney damage are reported to be induced by renin–angiotensin system (RAS) activation or Angiotensin II type I receptor (AT1R) and endothelin receptors [19], i.e., ET receptor type A and type B [147,148]. The activation of these receptors contributes to nephrotoxicity by increasing microvascular pressure and/or destruction of podocytes. Activation of these receptors leads to kidney damage by increasing microvascular pressure, podocyte autophagy, promoting oxidative stress and NFκB-p65 activation; which in turn trigger the release of inflammatory mediators like IFNγ, IL-6, and prostaglandins [149]. Ameliorative effect of naringenin in different drug-induced nephrotoxicity models has been reported. The Western blotting technique of protein analysis in rats suffering from daunorubicin-induced kidney damage has revealed upregulation of PPARγ and attenuation of AT1R, ETAR, p-NFκB p65, and p-ERK1/2 signaling pathways, thereby reducing oxidative stress and inflammation following naringenin treatment. Histopathological analysis of the kidney has also shown improvement in fibrotic lesions in a naringenin-administered group [150].

Additionally, involvement of peroxisome proliferator-activated receptors PPARγ in NF-kB and MAPK signaling in drug-induced renal toxicity in a rodent model has been reported [151]. Moreover, a marked reduction in levels of interleukin-8, serum creatinine level, and nitric oxide [22] level and reduced GPx activity after naringenin administration in gentamicin-treated rats have been reported [152]. Drug-induced nephrotoxicity is associated with the release of kidney injury molecule-1 (KIM-1) and IL-8, which are the kidney damage biomarkers. KIM-1, a glycoprotein, acts as a receptor for phosphatidylserine present on the cells undergoing apoptosis for phagocytosis [153], while IL-8 is an endothelial-derived cytokine responsible for oxidative burst and chemotaxis of neutrophils at the site of inflammation or injury. Immunohistochemical examinations of naringenin-treated tissues have shown significant downregulation of different biomarkers, including VEGF, iNOS, and caspase-9; in contrast, there is enhanced expression of survivin in the renal tissue [152]. Survivin is an anti-apoptotic factor that suppresses apoptosis by either inhibiting activation of caspase-9 [154] or by preventing cytochrome-c release via inhibition of Fas/Fas ligand stimulation [151]. Naringenin has also shown protective action against cisplatin-induced renal toxicity in a rat model [155], cadmium-mediated oxidative kidney impairment [98], and acute renal toxicity induced by carbon tetrachloride [96].

### 3.7. Gastroprotective Action

Because of the highly permeable nature of naringenin, it is absorbed throughout the gastrointestinal tract, largely in the small intestine via passive diffusion [156]. The gastroprotective effect of naringenin may be attributed to its anti-inflammatory activities, which is primarily achieved through inhibition of activator protein-1 and NF-κB signaling and activation of P38 MAPK and ERK 5 [157]. Over time, IBDs are known to progress to more severe forms like ulcerative colitis [156] and Crohn’s disease (CD). In such cases, treatment with naringenin has revealed anti-ulcerogenic effects in the form of decline in tissue ulceration, inflammation, and necrosis of the colon [158]. In addition, beneficial effects of naringenin may also be accomplished through suppression of pro-inflammatory markers like iNOS, reduction of inflammatory cytokines, diminished TLR4 mRNA, and protein concentration in mucosa [159].

Naringenin is activated by interaction with serotonin (5-HT4) receptor antagonists, which in turn increase the colonic contractions, enabling the process of gastric emptying [160]. Certain endogenous molecules like Motilin and Ghrelin released from the stomach, small intestines, and pancreas stimulate GI motility through their action on distinctive receptors [161]. Naringenin has been reported to function as an agonist to Ghrelin receptors, thereby improving various GI disturbances [162]. In SGC-7901 cell lines, naringenin exhibited induction of apoptosis along with suppression of proliferation, migration, and invasion. The possible reason may be the inhibition of AKT phosphorylation and decline in expression of target molecules by naringenin [163]. Zhang et al. have reported that naringenin suppressed the growth of stomach cancer cells in SGC-7901 cell lines [164].

### 3.8. Role of Naringenin in Pulmonary Diseases

Lung diseases such as asthma, chronic obstructive pulmonary disease (COPD), lung cancer, and pulmonary fibrosis are chronic airway inflammatory diseases (CAID) characterized by inflammatory response by the respiratory tract against certain external agents, including pathogens, smoke, and dust particles, resulting in increased production of inflammatory cells, mucous production, and inflammatory mediators causing impaired lung function. The inflammatory response in CAID is believed to be mediated by numerous inflammatory factors such as inflammatory cytokines, interleukins, glucocorticoid receptors, tumor necrosis factors (TNF-a), and nuclear factor kappa B (NF-kB). These inflammatory factors result in elevated oxidative stress, which stimulates immune responses, promotes hyper-responsiveness of the bronchi, and increases the secretion of mucin [165].

It has been reported that naringenin inhibits the inflammatory pathway involved in CAID by inhibiting NF-kB transcription activity [166]. Further, naringenin inhibits the inflammatory response in the respiratory tract initiated by microbial products involving pro-inflammatory cytokines, IL-1, NF-kB, and TNF-a. Naringenin inhibits the degradation of IkB that is bound to the p50/p65 subunit of NF-kB and thereby inhibits NF-kB activity [165,167]. Furthermore, numerous in vivo studies have shown the therapeutic effect of naringenin in airway inflammatory diseases [165].

### 3.9. Antimicrobial Action

Presently, natural products have drawn attention because of their potent antimicrobial activities and fewer side effects than chemically synthesized compounds. Natural products are considered safe and are advantageous over antibiotics because of the lesser chances of drug resistance associated with them. It is less likely for bacterial strains to develop resistance against a compound of natural origin because of their multiple mechanisms of action on bacterial cells. Natural products exert their antibacterial activity by interaction with the bacterial membrane and disruption of its integrity, inhibition of enzymes necessary for bacterial membrane synthesis, anti-biofilm activity, or inhibition of auto inducer-mediated cell–cell signaling [168].

Naringenin possesses a wide spectrum of antimicrobial activities, including antifungal and antibacterial [169]. Naringenin is found to possess potent activity against Methicillin-resistant *Staphylococcus aureus* [170] and resistant strains of *Helicobacter pylori* [171]. Naringenin exhibits its antibacterial action by inhibiting the autoinducer-mediated cell signaling pathway [172].

## 4. Conclusions and Future Perspective

In the present review, we have presented broad pharmacological aspects of naringenin reflected from the latest research. Evidence from collected literature has revealed that naringenin has several notable pharmacological properties, e.g., antioxidant, anti-inflammatory, antimicrobial, anti-diabetic, and anticancer. There are many molecular mechanisms fundamental to the pleotropic activities of naringenin, which involve the amalgamation of cellular signaling pathways at numerous levels of different diseases. Despite its tremendous potential in augmenting different diseases, naringenin is limited by a bioavailability problem, like other polyphenols. Methods to enhance the bioavailability of naringenin must be investigated to enhance the efficiency of naringenin as a therapeutic. Further active research is warranted to discover the promising therapeutic potentials of naringenin with respect to the promotion of better human health. Parallel research is also needed to enhance its bioavailability, which is instrumental in exerting the effective pharmacological effects of naringenin.
